# Two Cases With Atypical Presentation of Intestinal Malrotation During Adulthood

**DOI:** 10.1155/cris/9915368

**Published:** 2025-05-13

**Authors:** Aaron Womer, Vaibhav Duggal, Charles E. Thompson

**Affiliations:** Department of General Surgery, Icahn School of Medicine at Mount Sinai, South Nassau, Oceanside, New York, USA

## Abstract

Intestinal malrotation is often considered a disease of the newborn. It involves the failure of the 270° counterclockwise rotation of the midgut during embryonic development. Patients typically present with symptoms such as bilious vomiting and can further be diagnosed through imaging. The complications of intestinal malrotation include midgut volvulus which can cause ischemia of the intestine. In order to prevent this feared complication and treat malrotation, the four-step Ladd's procedure was developed. Proficiency of the procedure is more common among pediatric surgeons due to the higher incidence rate of malrotation; however, it can occur at any age. Adult presentations are reported to account for only 0.2%–0.5% of all cases of intestinal malrotation. Due to that, adult general surgeons are less likely to encounter the pathology and need to perform a Ladd's procedure. However, with the consequences being so dire, the ability to perform Ladd's procedure remains important in all general surgeons. In this report, we present two cases of intestinal malrotation in adults managed with Ladd's procedure and emphasize the importance with familiarity of both the pathology and procedure.

## 1. Introduction

Intestinal malrotation is considered a disease of the newborn, with 90% of cases diagnosed during the first year of life. It is defined by the failure of the 270° counterclockwise rotation of the midgut about the superior mesenteric pole during embryonic development [1]. Malrotation is a rare condition that has an incidence of 1 in 500 live births, but only 1 in 6000 newborn cases are symptomatic [2]. Adult presentations are even more uncommon and have been reported to account for only 0.2%–0.5% of cases, thus requiring a greater index of suspicion [3]. Presentation typically includes bilious vomiting, warranting further evaluation with imaging. Adult presentations may be an incidental finding on radiologic studies or found due to chronic nonspecific complaints. In a study done with 82 adult patients, Nehra et al. reported abdominal pain to be the most common complaint, followed by emesis and then nausea [3]. Diagnostic CT imaging may show the superior mesenteric artery to the right of the superior mesenteric vein, small bowel loops in the right upper abdomen, lack of visualization of the cecum in the right iliac fossa, or the duodenojejunal flexure on the right [4].

**Figure 1:**
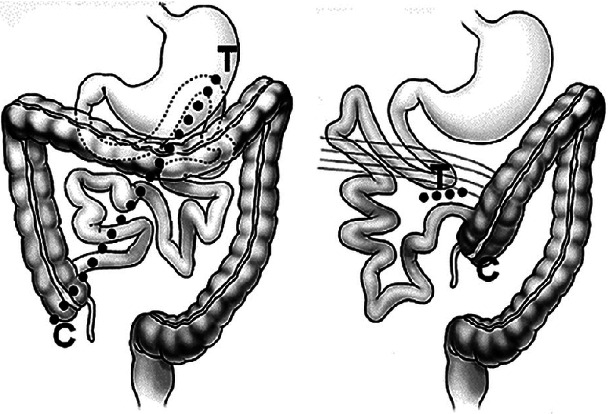
Intestinal rotation: (A) results of normal intestinal rotation, leading to a broad mesentery, denoted by the dotted line, splayed between the ligament of Treitz (T) and the cecum (C). (B) Results of intestinal malrotation, with a narrow-based mesentery (short dotted line) due to close proximity of the ligament of Treitz and the cecum [5].

The embryologic process of midgut rotation involves a 270° counterclockwise rotation about the mesenteric axis. During weeks 11 and 12 of development, the final fixation phase occurs. Any aberrance in the normal rotational process may result in findings consistent with rotational abnormality. Malrotation often presents with a Ligament of Treitz that is in close proximity to the cecum, resulting in a narrow mesentery (Figure 1). A common hallmark of malrotation is the formation of abnormal fibrous adhesions, known as Ladd's bands, due to abnormal fixation of the viscera to the lateral abdominal wall. Normal rotation results in a wide mesenteric base that extends from the ileocecal valve to the Ligament of Treitz. Malrotation is associated with a narrow mesenteric base, which makes the midgut more susceptible to volvulus [6].

Midgut volvulus is the most feared complication of intestinal malrotation, which can progress to ischemia of the intestine requiring resection. In 1936, surgical treatment of intestinal malrotation and midgut volvulus was defined by one of the founders of pediatric surgery, William E. Ladd. Ladd's procedure consists of four major steps. It begins with a counterclockwise detorsion of the intestines, followed by dissection of the Ladd's bands to relieve obstruction. The next step involves broadening the small intestine mesentery to prevent recurrent volvulus. The duodenum and jejunum are repositioned to the right and the colon to the left. The procedure is completed with an appendectomy to prevent future diagnostic dilemmas related to a left-sided appendix, and the cecum is positioned on the left [7]. Throughout the procedure, careful consideration must be made to avoid inadvertently ligating the colonic mesentery due to abnormal anatomy. While Ladd's procedure is the gold standard for the treatment of malrotation in children, many general surgeons may be unfamiliar with the atypical anatomy pertaining to this procedure [8]. The decision to approach Ladd's procedure laparoscopically or open will depend on patient factors as well as surgeons' familiarity with both approaches.

Our case report presents two cases of intestinal malrotation in adults with atypical presentations managed by Ladd's procedure.

## 2. Case Presentation 1

A 56-year-old woman with a history of GERD, symptomatic gallstones, and no prior abdominal surgery presented to the ED with complaints of severe epigastric abdominal pain that awoke her from sleep that morning, as well as nonbilious emesis.

Abdominal examination was notable for slight distention and mild tenderness of the epigastric region. There were no signs of peritonitis. The rest of the physical exam was benign.

**Figure 2:**
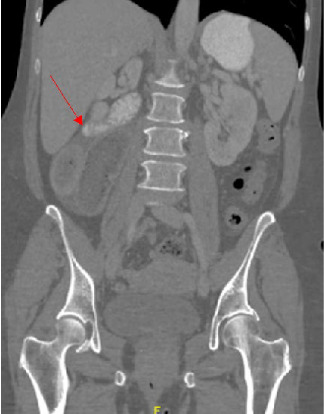
CT showing rightward sweeping duodenum (arrow) leading to jejunum without crossing the midline.

**Figure 3:**
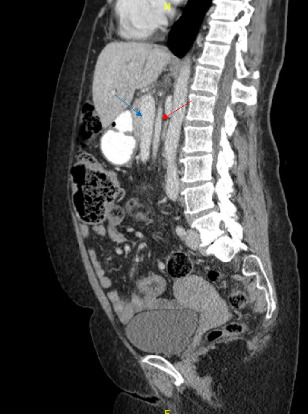
Anomalous position of the mesenteric vessels with SMA (red arrow) posterior to the SMV (blue arrow).

**Figure 4:**
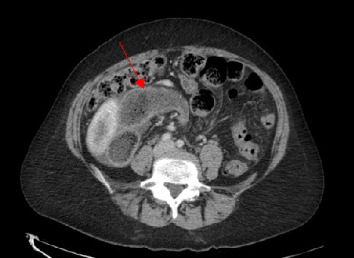
Bowel volvulus, thickened bowel wall, and fecalization of the small bowel contents (arrow).

The patient's blood work was unremarkable besides neutrophilia of 79.8%. A CT abdomen and pelvis showed signs of intestinal malrotation (Figures 2 and 3), a high-grade small bowel obstruction with fecalization of the small bowel contents (Figure 4), and moderate gastric dilation seen in the fundus. An abrupt transition was visualized in the right lower quadrant. Mesenteric fat stranding and ascites were concerning for possible ischemia. The cecum and appendix were seen left of the midline, and the duodenum was never seen crossing the midline (Figure 2). A diagnosis of intestinal malrotation with high-grade small bowel obstruction was made, and the patient was taken to the operating room for a diagnostic laparoscopy and possible bowel resection.

The patient was brought to the operating room, and under general anesthesia, a diagnostic laparoscopy was performed. After prepping and draping the abdomen, a 5 mm incision was made at Palmer's point, where pneumoperitoneum was achieved at 15 mmHg utilizing a Veress needle. The abdominal cavity was entered under direct laparoscopic vision of the left upper quadrant. Additional 5 mm XCEL trocars were then placed in the left mid abdomen as well as the left lower quadrant. Upon exploration, the small bowel appeared to be towards the right abdominopelvic cavity, while the transverse colon was confined to the right upper quadrant. The cecum was found medialized to the left side. Dense adhesive bands were noted over the cecum and colon extending across the mesentery to the abdominal wall. The duodenum was dilated due to compression by Ladd's bands anteriorly, however no bowel compromise was noted. A modified Ladd's procedure was conducted, as detorsion of volvulus was not necessary. We began with extensive lysis of adhesions using a Maryland LigaSure, including transection of Ladd's bands and full lateral mobilization of the right colon. During the dissection, some of Ladd's bands were found to be producing external compression on the duodenum, which was alleviated by the transection of those bands. The entirety of the small bowel was run twice. There was no volvulus present. Following the lysis of adhesions, the duodenojejunal junction was noted to be free of adhesions, coursing from the right upper quadrant directly inferiorly into the right abdomen. The mesentery of the bowel was flattened, resulting in the small bowel resting in the right side of the abdomen and the cecum and colon resting in the left hemiabdomen. An appendectomy was performed to conclude the operation.

The patient's postoperative course was uneventful, as there were no significant complications. The patient was followed for 6 months and was doing well. She was noted to have a resolution of obstructive symptoms and improvement in her abdominal pain.

## 3. Case Presentation 2

An 81-year-old male presented to the ED with complaints of severe abdominal pain since that morning associated with nausea and vomiting. He also reported diarrhea and vomitus that was a dark coffee ground color.

His past medical history is significant for melanoma of the eye with metastasis to the spleen and liver. He also has a history of congestive heart failure, hypertension, and CABG managed with aspirin. His surgical history is also significant for an appendectomy.

The patient appeared to be in distress due to pain, was tachycardic with a heart rate of 108 bpm, and had an elevated blood pressure of 162/80. On physical exam, the patient had mild distension, bilateral lower quadrant tenderness, and voluntary guarding.

**Figure 5:**
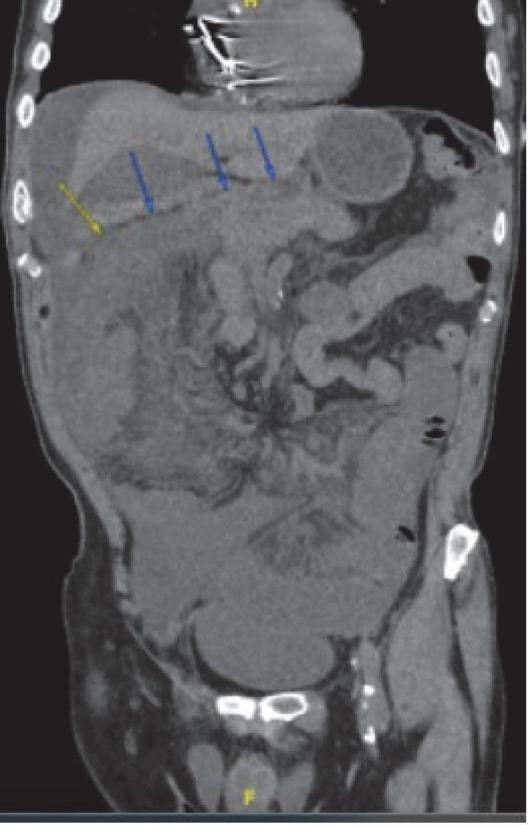
CT showing duodenojejunal junction in the right hemi-abdomen (arrows).

**Figure 6:**
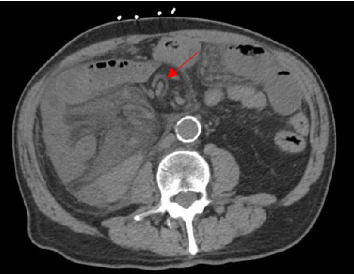
Left abdomen with diffuse mesenteric edema, bowel wall thickening, and mesenteric swirl (arrow).

Blood work was notable for a WBC count of 15.1k, creatinine of 1.61 mg/dL, elevated lactate, and metabolic acidosis. A CT abdomen pelvis was performed without IV contrast due to acute renal injury. Dilated loops of bowel were seen within the left hemiabdomen, indicating bowel obstruction alongside moderate gastric dilation, slightly obscured by the patient's significant mesenteric opacification. Configuration of the small bowel within the right hemiabdomen suggested an internal hernia as the etiology (Figure 5). Diffuse mesenteric edema of the small bowel in the right hemiabdomen was visualized, as well as bowel wall thickening, concerning bowel ischemia (Figure 6). The patient was taken for exploratory surgery due to concerns about intestinal ischemia and obstruction.

The patient was brought to the operating room for diagnostic laparoscopy. Upon entry to the abdomen, there was found to be a paucity of small bowel contents in the mid-abdomen, and cloudy ascites were present. The cecum was found in the right upper quadrant, and a thick band of tissue from the right abdominal wall spanned across the cecum to the duodenum and mid-transverse colon. The visible small bowel appeared purple and dusky and was seen herniating from a space inferior to the cecum. The mesentery of the proximal small bowel was noted to be herniating posterior to the mesentery of the right colon. These findings were consistent with a right paraduodenal hernia. The Ligament of Treitz was not able to be located, and multiple attempts at reducing the volvulus were unsuccessful using a laparoscopic technique, so a laparotomy was performed.

Open exploration confirmed an internal right paraduodenal hernia, with the majority of the small bowel herniating from a defect posterior to the mesentery of the cecum. Much of the small bowel was obscured from view, as it was confined in the internal hernia space posterior to the mesentery of the right colon. Congenital malrotation was also confirmed as the duodenojejunal junction was located solely in the right upper quadrant, not passing midline. Thick fibrous Ladd's bands were identified extending from the right abdominal wall to the cecum and duodenum. These bands were transected, and the right colon was fully mobilized, thus reducing the paraduodenal hernia, which was posterior to the mesentery of the right colon. Volvulus of the small bowel was reduced with counterclockwise rotation of the small bowel mesentery. Inspection of the small after detorsion showed a pink healthy bowel and resolution of the previous ischemic appearance. The colon was repositioned to the left hemiabdomen and the small bowel to the right, resulting in a broad, flat, common mesentery. Fibrous bands oriented transversely in the small bowel mesentery were divided, thus widening the small bowel mesentery. Surgical absence of the appendix was confirmed by examining the cecum. Skin and fascia were closed at the end of the procedure, and the patient was extubated.

His postoperative course was complicated by a limited episode of hypertensive emergency resulting in headache but no lasting neurological consequence. Bowel function returned and his diet was advanced slowly as tolerated. The patient was deemed stable for discharge and instructed to follow-up in 1–2 weeks as an outpatient.

## 4. Discussion

Intestinal malrotation is a congenital disorder that often leads to complications presenting in early childhood. However, these acute and life-threatening complications rarely present during adulthood among patients who were not previously known to have intestinal malrotation [3]. Acute presentation in adulthood may be due to obstruction, internal hernia, or ischemia. Many patients presenting with acute surgical complications of malrotation were previously misdiagnosed with nonsurgical disorders of chronic abdominal pain [8]. While adult onset malrotation is uncommon, it's vital for surgeons to understand the pathology alongside its treatment due to the potential for a life-threatening complication, midgut volvulus. The condition can lead to obstruction and compromised blood flow to the bowel, resulting in potential ischemia and bowel necrosis [9]. Early recognition is crucial, so physicians should maintain a high index of suspicion while examining the patient. Signs of acute illness, including a peritonitic abdomen and hemodynamic instability alongside potential imaging demonstrating whirlpool signs (clockwise twisting of vessels around the mesentery) or even free air due to perforation, should be addressed promptly [9]. Surgical intervention must be done in a timely fashion if required.

Here, we present two cases of adult patients presenting with acute manifestations of intestinal malrotation. Both were treated with modification of Ladd's procedure. The first patient was a middle-aged woman with chronic abdominal pain previously attributed to gallstones who presented with acute small bowel obstruction due to malrotation. Ladd's procedure was performed with transection of Ladd's bands, which were noted to be compressing the duodenum, however reduction of volvulus was not necessary. The second patient was an elderly man with previous abdominal surgery who presented with a symptomatic paraduodenal hernia associated as well as undiagnosed intestinal malrotation. His malrotation was classified as incomplete intestinal rotation, as the cecum was located in the right upper abdomen, and there was a right-side duodenojejunal junction. His previous appendectomy demonstrates findings that ~20% of adults with malrotation and midgut volvulus have had previous abdominal surgery without a diagnosis of malrotation [8]. Ladd's procedure was performed along with the reduction of the right paraduodenal hernia, which was accomplished by mobilizing the right colon. Both patients were treated successfully using a modified Ladd's procedure, one laparoscopic and one with laparotomy. Neither patient required bowel resection. At the time of follow-up, both patients were found to have improvement in abdominal pain and resolution of obstructive symptoms.

Indications to perform Ladd's procedure include any patient with complications of malrotation such as volvulus or obstruction. When performing the procedure, either a laparoscopic or an open approach can be used. Each approach has its benefits and faults, so there are a few things to consider. The first of which is surgical comfort. In order to perform a laparoscopic Ladd's procedure safely the attending surgeon must be able and confident in their laparoscopic technique. Next revolves around the specific case itself. Cases with significant interstitial dilation may present a barrier to the laparoscopic approach, as there may be limited working space in the peritoneal cavity. Thus, an open approach may be best suited. As with many laparoscopic to open procedure comparisons, the minimally invasive approach would have less morbidity and likely a shorter length of stay. In a study of 226 patients, with 40% undergoing a laparoscopic Ladd's procedure, it was concluded that they were less likely to develop a postoperative small bowel obstruction compared to their open-approach counterparts. However, due to the development of strictures in the laparoscopic approach patients, there was no overall difference reported in rates of reoperation [10]. Other literature has been published demonstrating the feasibility and effectiveness of a laparoscopic alternative to the open approach. One study followed seven patients who underwent laparoscopic Ladd's procedures with all seven being discharged within three postoperative days and no early complications. Overall, 71% of those patients maintained substantial improvement in abdominal discomfort months after surgery [11]. With literature supporting the option of minimally invasive Ladd's procedures, the choice now comes down to the attending surgeon and their assessment of the case as a whole.

General surgeons should be familiar with intestinal malrotation and its associated complications, as these rarely present as acute surgical issues among adult patients. Many patients with acute complications of intestinal malrotation are misdiagnosed with chronic abdominal pain disorders, even despite previous abdominal surgery. These cases demonstrate the potential for general surgeons to encounter intestinal malrotation in adults and have the need to perform Ladd's procedure. General surgeons should be familiar with Ladd's procedure because it offers an opportunity to treat and prevent future episodes of midgut volvulus at the time of surgical exploration.

## Data Availability

Data sharing is not applicable to this article as no new data were created or analyzed in this study.

## References

[1] Synder W. H., Chaffin L. (1954). Embryology and Pathology of the Intestinal Tract: Presentation of 40 Cases of Malrotation. *Annals of Surgery*.

[2] Dehaini H., Nasser Eldine R., Doughan S. (2020). Presentation of Intestinal Malrotation and Midgut Volvulus in Adults Case Report and Literature Review. *International Journal of Surgery Case Reports*.

[3] Ferreira M. S., Simões J., Folgado A. (2020). Recurrent Midgut Volvulus in an Adult Patient—The Case for Pexy? A Case Report and Review of the Literature. *International Journal of Surgery Case Reports*.

[4] Eccleston J. L., Su H., Ling A., Heller T., Koh C. (2016). Gastrointestinal: Adult Presentation of Intestinal Malrotation. *Journal of Gastroenterology and Hepatology*.

[5] Vidal E. A., Rendon F. A., Zambrano T. A. (2016). Intestinal Malrotation in Patients Undergoing Bariatric Surgery. *Arquivos Brasileiros de Cirurgia Digestiva*.

[6] Perez Galaz F., Moedano Rico K., Pérez Tristán F. A., Acuña Macouzet A., Jafif Cojab M. (2020). Midgut Volvulus Caused by Intestinal Malrotation; A Rare Cause of Acute Abdomen in Adults Case Report. *International Journal of Surgery Case Reports*.

[7] Bhatia S., Jain S., Singh C. B., Bains L., Kaushik R., Gowda N. S. (2018). Malrotation of the Gut in Adults: An Often Forgotten Entity. *Cureus*.

[8] Neville J. J., Gallagher J., Mitra A., Sheth H. (2020). Adult Presentations of Congenital Midgut Malrotation: A Systematic Review. *World Journal of Surgery*.

[9] Coste A. H., Anand S., Nada H., Ahmad H. (2020). Midgut Volvulus. https://www.ncbi.nlm.nih.gov/books/NBK441962/.

[10] Johnston W. R., Hwang R., Mattei P. (2024). Laparoscopic Versus Open Ladd Procedure for Midgut Malrotation. *Journal of Pediatric Surgery*.

[11] Seymour N. E., Andersen D. K. (2005). Laparoscopic Treatment of Intestinal Malrotation in Adults. *JSLS: Journal of the Society of Laparoendoscopic Surgeons*.

